# Plant-derived tormentic acid alters the gut microbiota of the silkworm (*Bombyx mori*)

**DOI:** 10.1038/s41598-022-17478-4

**Published:** 2022-07-29

**Authors:** Veysel Bay, Seray Gür, Oğuz Bayraktar

**Affiliations:** 1 Department of Animal Science, Faculty of Agriculture, Ege University, 35100 Bornova, Izmir Turkey; 2 Department of Bioengineering, Faculty of Engineering, Ege University, 35100 Bornova, Izmir Turkey

**Keywords:** Metagenomics, Entomology

## Abstract

In recent years, phytochemicals have started to attract more attention due to their contribution to health and bioactivity. Microorganisms in the intestines of organisms contribute to the processing, function, and biotransformation of these substances. The silkworm (*Bombyx mori*) is one of the organisms used for the biotransformation of phytochemicals due to its controlled reproduction and liability to microbial manipulation. In this study, a bioactive compound, tormentic acid (TA), extracted from *Sarcopoterium spinosum* was used in the silkworm diet, and the alterations of intestinal microbiota of the silkworm were assessed. To do this, silkworms were fed on a diet with various tormentic acid content, and 16S metagenomic analysis was performed to determine the alterations in the gut microbiota profile of these organisms. Diet with different TA content did not cause a change in the bacterial diversity of the samples. A more detailed comparison between different feeding groups indicated increased abundance of bacteria associated with health, i.e., *Intestinibacter* spp., *Flavonifractor* spp., *Senegalimassilia* spp., through the utilization of bioactive substances such as flavonoids. In conclusion, it might be said that using TA as a supplementary product might help ameliorate the infected gut, promote the healthy gut, and relieve the undesirable effects of medicines on the gastrointestinal system.

## Introduction

Herbal natural compounds include phenolic compounds and secondary plant metabolites, generally known as phytochemicals. These compounds have started to gain significant importance with their positive effects against many diseases due to their bioactivity^1,2^. Most of the natural compounds undergo microbial changes, namely biotransformation, in the small intestine before being transformed in the liver^3^. Biotransformation processes using mammalian, microorganism, plant cells and insect cell cultures or enzymes isolated from these play an essential role in the discovery and development of new drug molecules^4–6^.

The silkworm (*Bombyx mori*), which feeds on mulberry (*Morus alba*) leaves, has been domesticated for thousands of years to produce economically important silk fibre^7^. Silkworm has provided a livelihood for humans for centuries and has been a highly productive source of biological and chemical substances^8,9^.

In addition to the benefits of silkworm itself, many compounds are produced from its excrement, which is the source of the treatment and food for other organisms^10–12^. Besides, it is possible to form bioactive compounds by feeding the silkworm with mulberry leaves enriched with different natural compounds by using the silkworm's biotransformation ability^13,14^.

Compounds that prevent liver damage, trigger tissue regeneration, or reduce the activity of toxic substances are called hepatoprotective agents. Some of the natural herbal compounds have antimicrobial, antidiabetic, antifungal, and hepatoprotective effects^15,16^. It is stated that tormentic acid (TA), one of the terpenes found in the content of *Sarcopoterium spinosum*, has the potential to be a hepatoprotective agent, as well as its effectiveness in type 2 diabetes^17,18^.

Many microorganisms live in the body of the silkworm. However, the microbiota of this insect model has not been well characterized to date^7,19^. With the advent of metagenomics technology, microbial communities of various species have been characterized and listed by genome studies on vertebrates and invertebrates^20^.

The silkworm gut has less microbial diversity compared to the other vertebrates due to the controlled microenvironment for growth and development and shorter lifespan. In addition, the silkworm microbiota must be able to tolerate the alkalinity up to pH > 10^21^, which adversely affects the microbiota of most vertebrates, and the change of the peritrophic matrix during moulting also limits the microbial growth^22^. In addition, the silkworm gut has low oxygen (O_2_) levels, so it only allows facultative anaerobic microorganisms to grow^23^**.**

Diet greatly impacts the gut microbiota profile of the organisms, and it is significantly associated with health and disease^24,25^. It is also possible to manipulate gut microbiota by using different diets^26–30^. Tormentic acid has been shown to take part in the treatment of bacterial disorders such as ulcerative colitis^31^, and periodontal disease^32^. Therefore, it could be a good candidate molecule for manipulation of the gut microbiota. In the present study, our aim was to investigate the effect of feeding silkworms with a diet, consisting of different tormentic acid content, on the microbiota profile of the silkworm gut.

## Results

Intestine samples were collected from silkworms fed on a diet with various tormentic acid content. The number of samples in each group is shown in Table 1.

**Table 1:** The number of samples in each group.

Group	TA_rich	TA_poor	Control	RawExtract
Number	6	3	4	2

**Figure 1:**
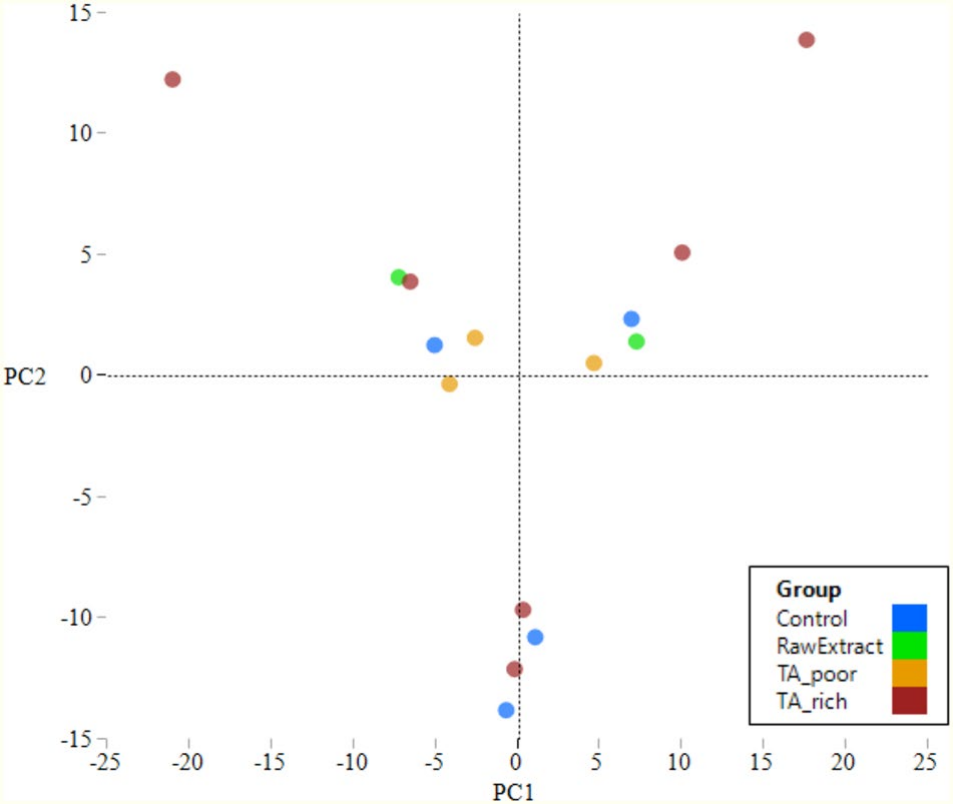
Scatterplot of the first 2 PCs calculated for the microbiota composition of the samples.

Shannon and Chao1 alpha-diversity indexes of the samples are presented in Table 2. ClustVis webtool^33^ was used to calculate principal components (PCs) and PC values were charted in scatterplots in JMP Pro 13. The microbiota composition of the samples did not indicate any clear clustering (Fig. 1).

**Table 2:** Shannon and Chao1 alpha-diversity indexes of the samples.

	Control [Q1:Q3] Median	TA_Poor [Q1:Q3] Median	TA_rich [Q1:Q3] Median	RawExtract [Q1:Q3] Median	*p v*alue
Shannon	[3.15:5.56] 5.46	[2.59:4.45] 4.02	[1.81:5.52] 4.22	[2.34:3.15] 2.75	0.674
Chao1	[136.71:178.62] 161.50	[101.36:182.61] 159.71	[169.00:228.33] 191.93	[169.71:202.00] 185.86	0.440

**Figure 2:**
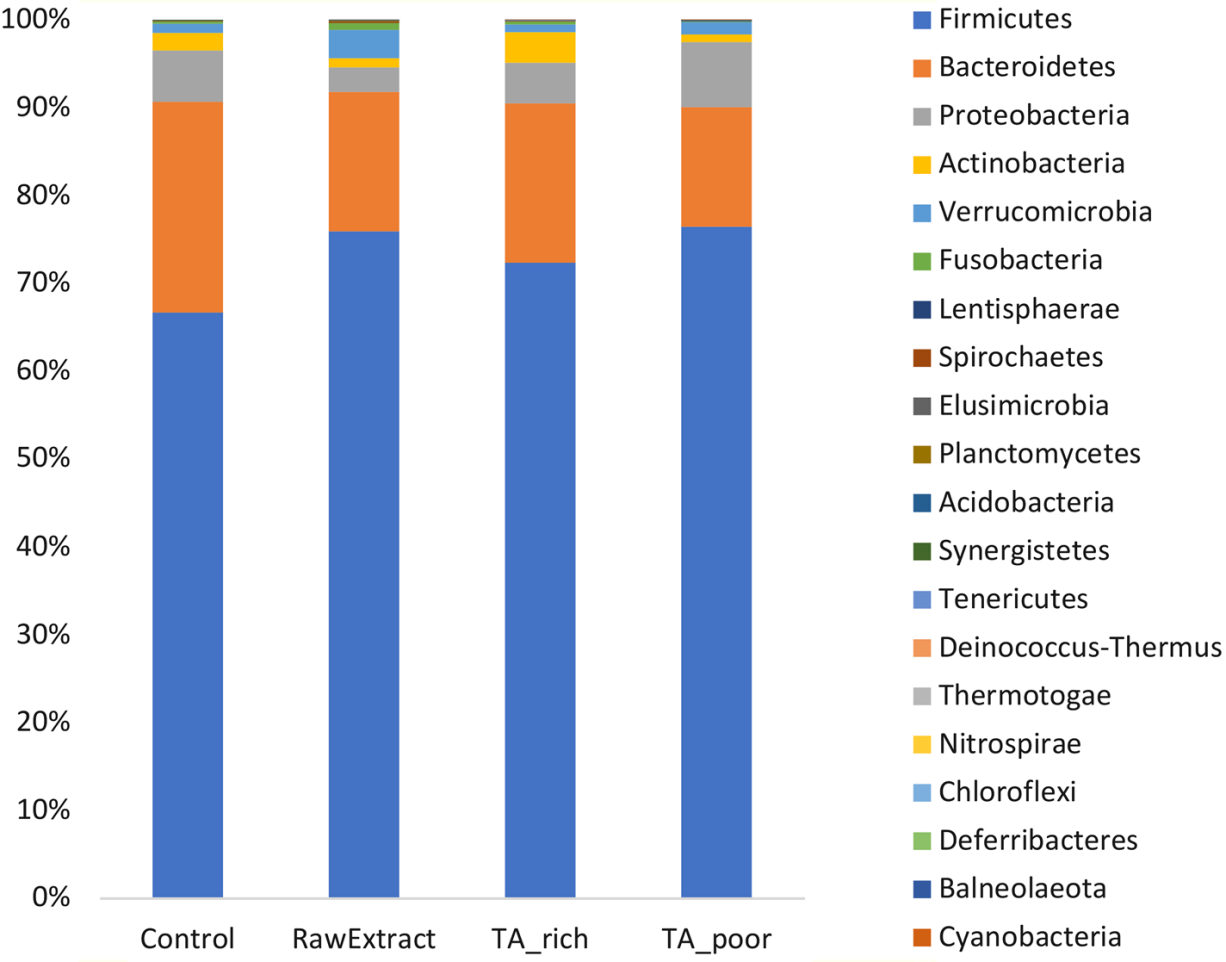
Relative abundances of bacterial phyla in all feeding groups.

In total, 20 phyla were detected, and relative abundances of these phyla were charted in Fig. 2. Firmicutes, Bacteroidetes, Proteobacteria, and Actinobacteria constitute more than 90% of all phyla for all the samples.

**Figure 3:**
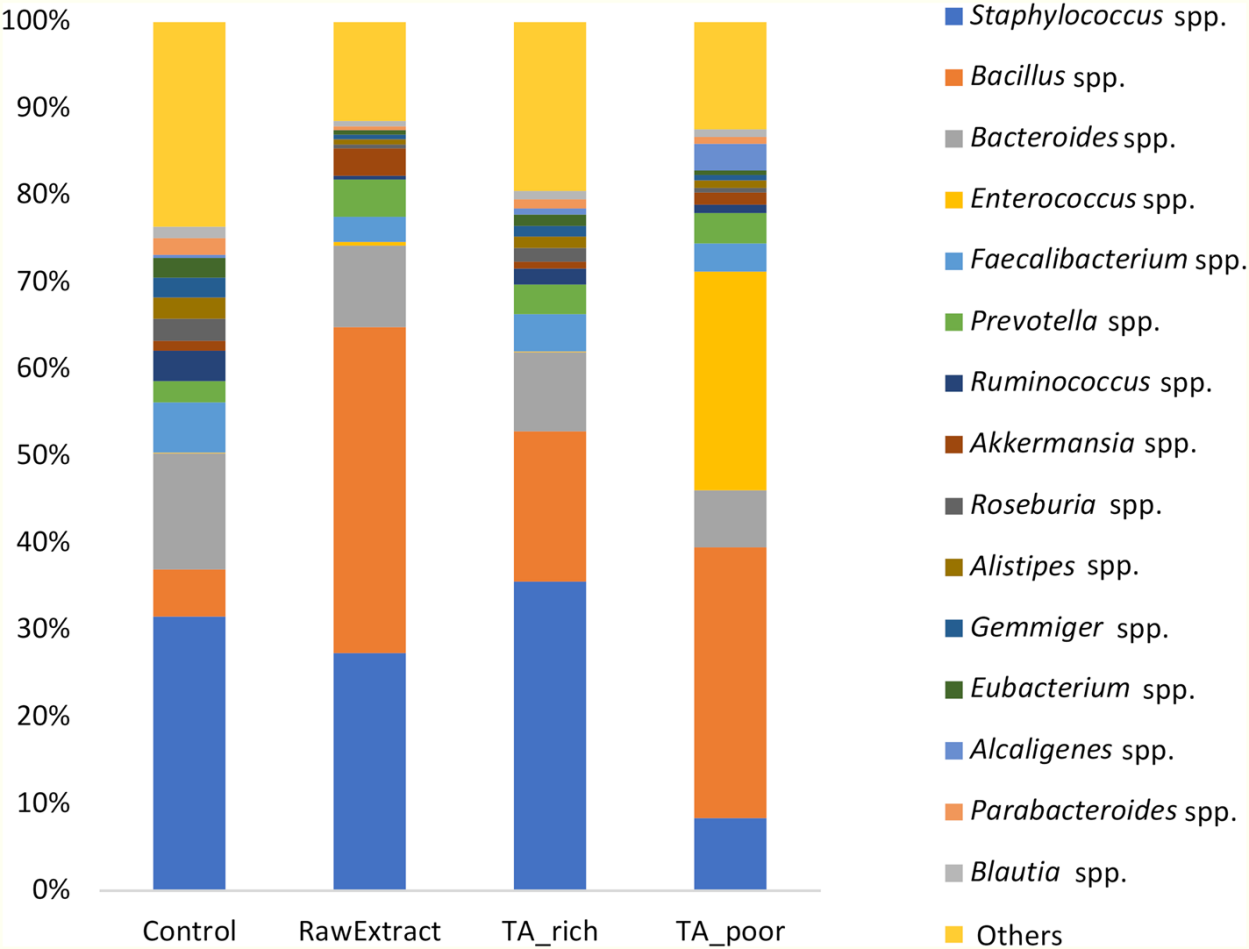
Relative abundances of the fifteen most prevalent genera in all feeding groups.

Figure 3 shows the relative abundances of the 15 most prevalent genera in all groups. *Staphylococcus* spp. constituted approximately 30% of all genera in Control, RawExtract and TA_rich feeding groups. TA_poor and RawExtract groups had relatively more *Bacillus* spp. compared to TA_rich and Control groups. The TA_poor group also had relatively more *Enterococcus* spp. compared to the other groups.

**Figure 4:**
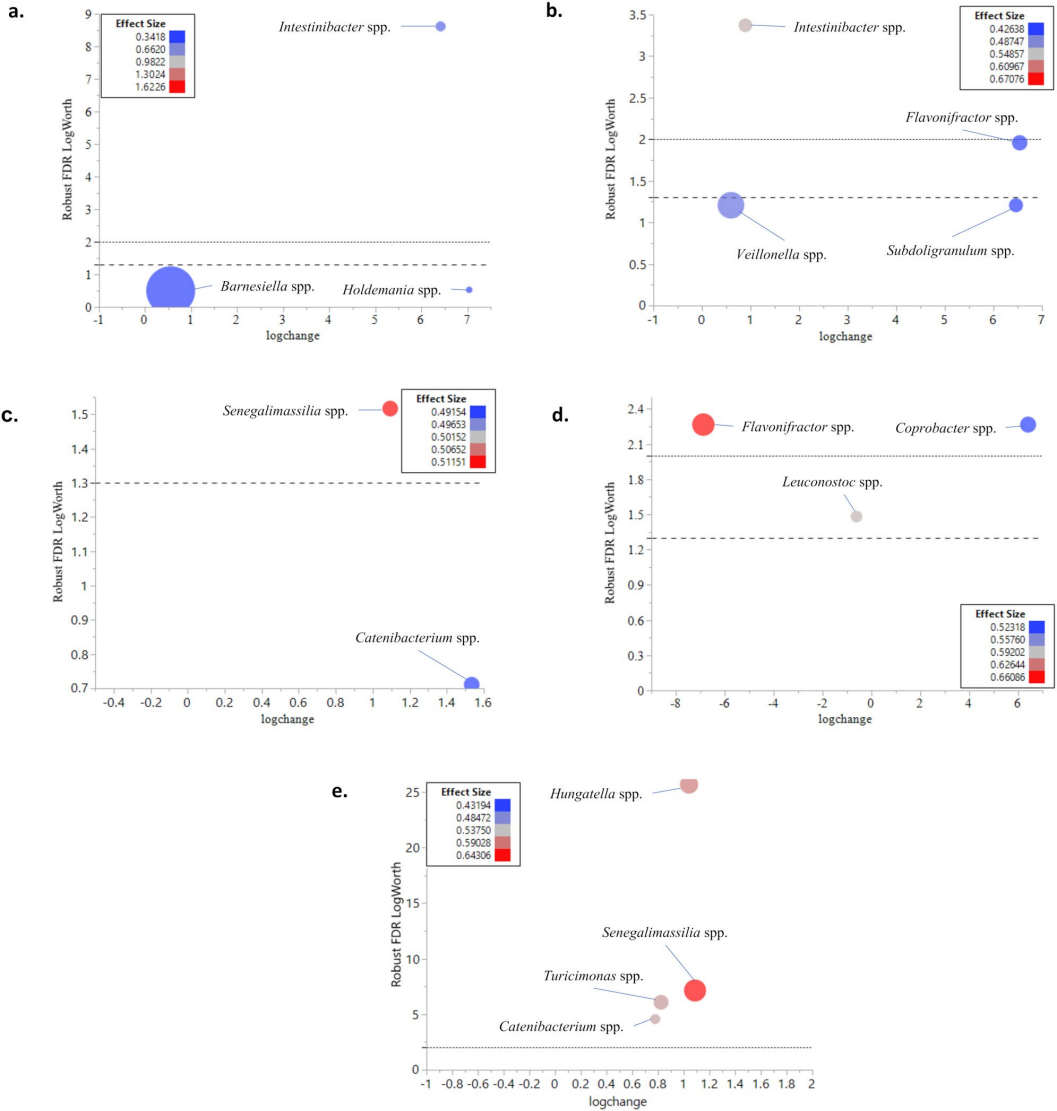
Comparison of the microbiota profiles of (**a**) TA_rich versus RawExtract, (**b**) TA_rich versus TA_poor, (**c**) TA_rich versus Control, (**d)** TA_poor versus RawExtract, (**d)** RawExtract versus Control feeding groups (line at 1.3 (- - -) = *p* value 0.05, line at 2 (----) = *p* value 0.01 adjusted for FDR) The log fold change in genera relative abundances in samples is plotted versus the corrected robust false discovery rate (FDR) LogWorth (i.e., log10P). The size of the circles represents the mean relative abundance of each genus, and colour represents the effect size.

The response screening analysis resulted in a more detailed analysis of the differences at the genus level between the groups. Based on the response screening analysis, *Intestinibacter* spp. were significantly more abundant in the gut microbiota of silkworms fed on a TA_rich diet compared to the ones fed on raw extract (Fig. 4a). TA_rich group samples also had significantly more prevalent *Intestinibacter* spp. when compared to the TA_poor group samples. Besides that, *Flavonifractor* spp. and some members of *Veillonella* spp. were significantly more prevalent in TA_rich group samples compared to the TA_poor group samples (Fig. 4b).

TA_rich group samples had significantly more abundant *Senegalimassilia* spp. compared to the Control group samples (Fig. 4c).

The silkworms fed on raw extract had significantly more abundant *Flavonifractor* spp. and *Leuconostoc* spp. in their gut compared to the ones fed on a TA_poor diet. However, TA_poor group samples had significantly more prevalent *Coprobacter* spp. compared to the Raw Extract group samples (Fig. 4d). There was no significant difference between the TA_poor and Control group samples (Supplementary Figure 2).

Raw Extract group samples had significantly more prevalent *Hungatella* spp., *Senegalimassilia* spp., *Turicimonas* spp., *Catenibacterium* spp. compared to the Control group samples (Fig. 4e).

## Discussion

In the present study, we have investigated the alterations in the gut microbiota profile of silkworms fed on diets comprising different TA content using 16S rRNA gene sequencing. TA, a terpene, is a bioactive molecule with the potential to be a hepatoprotective agent, and it is also effective in type 2 diabetes^17,18^. We have chosen the silkworm because it is an advantageous model organism due to its low gut microbial diversity compared to other organisms. Feeding silkworms with TA and evaluating changes in the gut microbiota profile of silkworms may provide insight into the effects of of terpenes in the gut. Although there was a certain difference in the microbiota profiles of the feeding groups, there was no significant difference in bacterial diversity of these groups. This situation might be caused by the controlled microenvironment in which silkworms are grown and the slight microbial changes that occur after feeding. In this study, fragments extracted from *Sarcopoterium spinosum* with different TA content were used in the diet of silkworms. The silkworm fed on TA_rich diet had significantly more *Intestinibacter* spp. compared to the ones fed on RawExtract and TA_poor diet. TA_rich group samples also had significantly more *Flavonifractor* spp. and some members of *Veillonella* spp. compared to TA_poor group samples. It was previously reported that the abundance of *Intestinibacter* spp. decreases and the abundance of *Escherichia* spp. increases in type 2 diabetes patients treated with metformin^34–36^. Metformin is an effective medicine widely used for type 2 diabetes, and one of the adverse effects of metformin is gastrointestinal disorders^37,38^ which could be associated with increased abundance of *Escherichia* spp^39^. Furthermore, current knowledge on the role of *Intestinibacter* spp. is scarce, but functional annotations suggest a role for these bacteria in mucus production through consumption of mucins^35,40^, which are helpful for immune and metabolic responses. *Flavonifractor* spp. are known for their role in the degradation of flavonoids^41,42^, which are bioactive molecules like terpenes^43^. *Flavonifractor* spp. have also been reported to increase with green tea consumption and promote recovery from acute colitis in mice^44^*. Veillonella* spp. are known as producers of propionate that supports health in the human gut^45^. TA_rich group samples had significantly more prevalent *Senegalimassilia* spp. compared to the control group. Like *Intestinibacter* spp., the abundance of *Senegalimassilia* spp. has been shown to decrease just after the metformin treatment. The relative abundance of *Senegalimassilia* spp has also been reported to be reduced in human polycystic ovarian syndrome cases^46^, and overweight children compared to the normal weight ones^47^. In conclusion, it can be said that TA, which is currently used in the treatment of type 2 diabetes, may have the potential to reverse the side effects of the widely used medication metformin. TA could also be given with metformin as a supplementary material to type 2 diabetes patients. To be able to assert this more precisely, further studies with a larger sample size and appropriate experimental design are required.

The relative abundance of *Flavonifractor* spp. was significantly higher in RawExtract samples compared to TA_poor samples. On the other hand, TA_poor samples had higher abundance of *Coprobacter* spp., and *Leuconostoc* spp. than RawExtract samples. *Coprobacter* spp. were shown to be positively associated with dietary uptake of polyphenols^48^, and *Leuconostoc* spp., members of lactic acid bacteria, have been shown to be associated with the production of extracellular polysaccharides^49^.

RawExtract group samples had a relatively higher abundance of *Hungatella* spp., *Senegalimassilia* spp., *Turicimonas* spp., *Catenibacterium* spp. compared to the Control group samples. Hungatella spp. play roles in converting carbohydrates into acetic acid, which is involved in ATP synthesis pathways^50^. *Catenibacterium* spp. were shown to be associated with the fermentation of fibre and production of short chain fatty acids, which promote gut health^51^. To date, there is no reported association between *Turicimonas* spp. and bioactive molecules.

In conclusion, the microbiota profile of *Bombyx mori* could be manipulated with the diet containing the terpene, TA. Our results indicated an increase in the relative abundance of bacteria associated with a healthy gut in TA-rich diets. Hence, it might be said that TA could be used as a supplementary product to ameliorate and stabilize the healthy gut. It could also be used as a reversal agent to alleviate the adverse effects of medicines. On the other hand, the sample size is a significant limitation for this study, but the results provide an insight into the microbiota changes in the case of TA supplementation to the silkworm diet. In brief, the results we report in this study, albeit on a small scale, may lead to potential future studies.

## Methods

### Sampling

*Sarcopoterium spinosum* plant was incubated at 60 °C for 6 h in 70% ethanol at a solid–liquid ratio of 1:20. The solvent was removed in the evaporator, and the extract was obtained in the water phase. A part of this extract was used in the diet of the silkworm in the RawExtract group. The remaining extract was taken into a separatory funnel and butanol was added. It was incubated until phase formation was observed, and then the water and butanol phases were separated. The butanol phase was taken into a 50 ml tube as TA_poor fraction and the remaining extract was evaporated to remove the butanol. The remaining phase was subjected to liquid–liquid extraction using ethyl acetate. The ethyl acetate phase was used as TA_rich fraction. These fractions were dissolved in 70% ethanol and sprayed on mulberry leaves. After the ethanol was evaporated, the mulberry leaves were used for feeding twice a day for 20–30 days.

Polyhybrid silkworm crosses with Japanese and Chinese origin provided by Koza Birlik company (Bursa, Turkey) were reared at 26 ± 2 °C with 75–85% humidity and regular daylight photoperiod. Silkworm in 5th instar were fed on mulberry leaves incubated with different fractions of *S. spinosum* extraction; RawExtract, TA_rich, TA_poor, and control. The silkworm larva was fixed with the help of a needle from the head and tail, and its shell was carefully cut to open the digestive tract. The gut tissue was separated and placed in a sterile 1.5 ml tube for gastrointestinal microbiota analysis.

### DNA extraction

Microbial DNA was extracted using the EurXGeneMATRIX Tissue and Bacterial DNA purification kit (EurX Ltd., Poland) and following the manufacturer’s instructions. The Qubit™ dsDNA HS Assay Kit (Thermo Fisher Scientific, Fair Lawn, NJ, USA) was used to measure DNA concentrations before PCR.

### 16S rRNA gene amplification, and sequencing

The 341F (Illumina_16S_341F 5′-TCG TCG GCA GCG TCA GAT GTG TAT AAG AGA CAG CCT ACG GGN GGC WG CAG), and 805R (Illumina_16S_805R 5′-GTC TCG TGG GCT CGG AGA TGT GTA TAA GAG ACA GGA CTA CHV GGG TAT CTA ATC C) universal primers with adapter sequences were used^52^ for amplification of the V3-V4 hypervariable region of 16S rRNA gene.

For the first step PCR, 5 μl of forward primer (1 μM), 5 μl of reverse primer (1 μM), 2.5 μl of microbial DNA (5 ng/μl) and 12.5 μl of 2X KAPA HotStart PCR Mix (Roche, Switzerland) were used at 95 °C initial denaturation for 3 min, followed by 25 cycles of 95 °C for 30 s, 55 °C for 30 s, and 72 °C for 30 s, and a final extension at 72 °C for 5 min. PCR products were cleaned up with AgencourtAMPure XP beads (Beckman Coulter Genomics, Fullerton, CA, USA) following the manufacturer’s protocol.

In a second PCR step, dual indices and Illumina sequencing adapters were attached using 5 μl of PCR product DNA, 5 μl of Illumina Nextera XT Index Primer 1 (N7xx), 5 μl of Nextera XT Index Primer 2 (S5xx), 25 μl of 2X KAPA HotStart PCR Mix, and 10 μl of nuclease free water with thermocycling at 95 °C for 3 min, followed by 8 cycles of 95 °C for 30 s, 55 °C for 30 s, and 72 °C for 30 s, and a final extension at 72 °C for 5 min. The final PCR products were cleaned with AgencourtAMPure XP beads, and final concentrations of samples were measured with Qubit™ dsDNA HS Assay Kit. Amplicon libraries was sequenced on a lane of the Illumina® MiSeq platform.

### Bioinformatics

Sequenced raw data was converted to FASTA format. Quality control of reads was performed using QIIME 2 software^53^. Reads with Phred scores lower than 20, primer and barcode sequences, and chimeric sequences were filtered out using DADA2 software^54^. Taxonomic assignment of each cluster was carried out using QIIME2 software to match a representative sequence from each OTU to a sequence from the GreenGenes database.

### Statistical analyses

The richness and evenness of the samples were analysed using Chao1 and Shannon diversity indexes. The indexes of groups were compared to each other using Kruskal–Wallis test. Beta-diversity of the samples were analysed and compared using the first three principal components. Mean relative abundances of the twenty phyla and fifteen genera were charted compared for each pair of groups. Microbiota profiles of each group were compared to each other as previously described^55^. Briefly, Logfold changes (Log10) in relative abundance of the genera were calculated for each group, and pairwise comparisons between groups were performed. Robust response screening analysis was performed in JMP Pro 13 (SAS Institute Inc., Cary, NC) in order to evaluate the differences in genera relative abundance between pairs of groups. A false discovery rate (FDR) correction was applied, and statistical significance was declared at FDR LogWorth of 1.3 (equivalent of a *p* value of 0.05), and 2 (equivalent of a *p* value of 0.01). Subsequently, the log fold change was plotted versus the Robust FDR LogWorth value using bubble plot graphs in JMPPro 13. Genera mean relative abundance defined the bubbles’ size, and the bubbles’ colouring indicated effect size.

## Supplementary Information

Supplementary Information.

## Data Availability

Sequences are available on the MG-RAST metagenomics analysis server at https://www.mg-rast.org/mgmain.html?mgpage=project&project=mgp101832. Raw data for this project were also deposited in the Bioproject database with the accession number PRJNA861165 (https://www.ncbi.nlm.nih.gov/bioproject/861165).
