# Supplementary material for: Plant-derived tormentic acid alters the gut microbiota of the silkworm (Bombyx mori)
Source: Sci Rep. 2022 Jul 29;12:13005. doi: 10.1038/s41598-022-17478-4 (PMC9338012; doi:10.1038/s41598-022-17478-4)
Supplement: Supplementary file 1 — Supplementary Information. [file 41598_2022_17478_MOESM1_ESM.docx]

**Plant-derived Tormentic Acid Alters the Gut Microbiota of the Silkworm (*Bombyx mori*)**

**Veysel Bay^1*^, Seray Gür^2^, Oğuz Bayraktar^2^**

**^1^** Department of Animal Science, Faculty of Agriculture, Ege University, 35100 Bornova, Izmir, Turkey

^2^ Department of Bioengineering, Faculty of Engineering, Ege University, 35100 Bornova, Izmir, Turkey

*Corresponding author

**Supplementary Tables**

**Supplementary Table 1. HPLC analysis and tormentic acid content results of S. spinosum extract and fractions**

| **Concentration** | **Sample** | **Tormentic Acid   (mg/100mg extract)** |  |
| --- | --- | --- | --- |
|  |  |  |  |
| 7mg/ml | Raw Extract | 1,6 |  |
| 7mg/ml | TA_poor | 0,3 |  |
| 7.1mg/ml | TA_rich | 31,3 |  |

**Supplementary Figures**


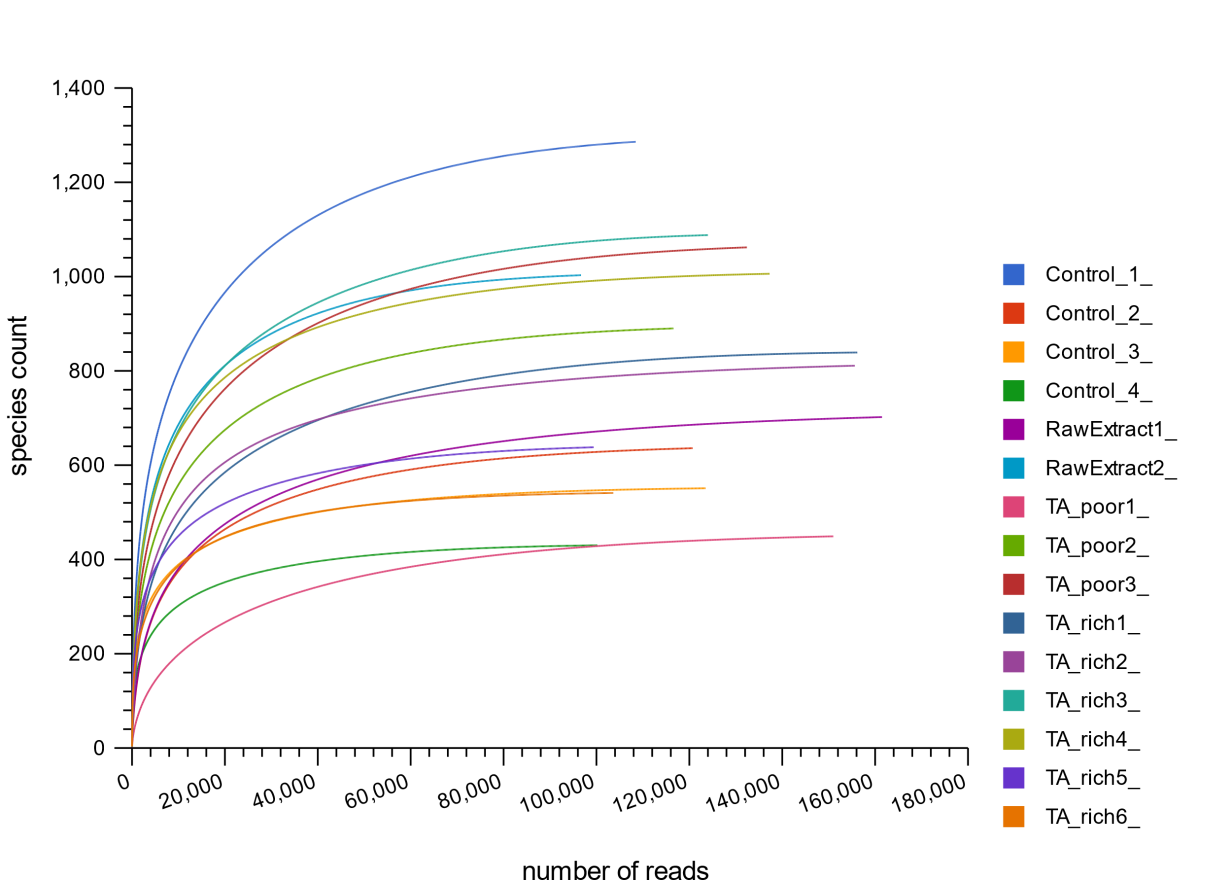


Supplementary Figure 1. The rarefaction curves of all samples


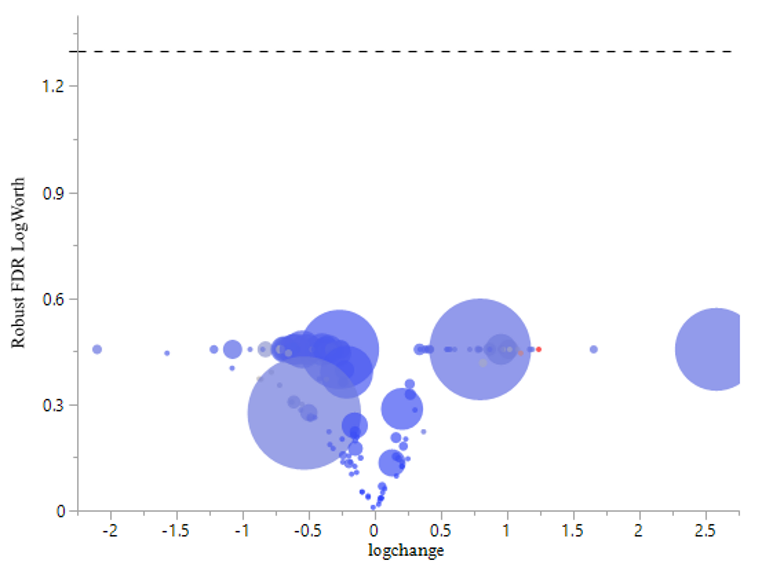


Supplementary Figure 2. Comparison of the microbiota profiles of TA_poor vs Control feeding groups (line at 1.3 (- - -) = *p*-value 0.05, line at 2 (----) = *p*-value 0.01 adjusted for FDR) The log fold change in genera relative abundances in samples from TA_poor group comparing to Control group samples is plotted versus the corrected robust false discovery rate (FDR) LogWorth (i.e., log10P). Size of the circles represents the mean relative abundance of each genus, and colour represents the effect size.
